# Rapid positioning of nasogastric tube by ultrasound in COVID-19 patients

**DOI:** 10.1186/s13054-020-03285-8

**Published:** 2020-09-22

**Authors:** Anyu Qian, Shanxiang Xu, Xiao Lu, Luping Tang, Mao Zhang, Xiao Chen

**Affiliations:** Department of Emergency Medicine, The Second Affiliated Hospital of Zhejiang University, School of Medicine, Hangzhou, 310009 China

**Keywords:** COVID-19, ultrasound, nasogastric tube

Dear editor,

It is well known that early enteral nutrition therapy is one of the basic management for critically ill COVID-19 patients. Nasogastric tube (NGT) is the most common access for enteral nutrition, and the correct positioning of NGT is a prerequisite. Otherwise, the malposition may result in severe complications, including asphyxia and pneumonia.

The pandemic of COVID-19 caused the overload of local medical service system, especially intensive care resources, such as ICU beds, ventilators, and intensivists. On February 14, a critical care team including 42 doctors, 123 nurses, and 6 logistical staffs from the Second Affiliated Hospital of Zhejiang University (SAHZU) was dispatched to Wuhan to take over a temporary ICU and admitted 61 critically ill patients with COVID-19. The positioning of NGT became one of big challenges. Bedside radiography which is the standard method of positioning NGT was not accessible in time in the temporary ICU. The traditional method is auscultating for sounds by stethoscope in the epigastrium while injecting air into NGT, but it is unreliable [1, 2]. Furthermore, the stethoscope is difficult to use due to the strict personal prevention of medical staff. Measuring PH value of gastric juice is an alternative method, but sometimes, it was not available, while monitoring end-tidal carbon dioxide by NGT can only exclude the malposition of NGT in airway [3, 4]. Some studies reported the role of ultrasound in positioning NGT [4], especially in settings where X-ray is not readily available, and ultrasound may be useful to detect misplaced gastric tubes [5]. Therefore, we tried to confirm the right place of NGT by ultrasound for these COVID-19 patients.

We introduced a specific procedure for rapid positioning NGT, based on the use of a portable ultrasound (M9, Mindray CO. LTD, China). The probe was oriented towards the left upper abdominal quadrant to visualize the gastric area. If 2 parallel hyperechogenic lines were seen through cardia, or ultrasound image showed dynamic hyperechogenic air area in the stomach when 20ml of air was injected through the NGT (Fig. 1), correct positions of NGT could be confirmed. Two other traditional methods were also combined, one was to place the end of the gastric tube in water and observe bubbles and the other was to observe gastric juice extraction. After confirming the right position of NGT by these three methods, we initiated enteral nutrition. We performed this procedure in 10 patients with COVID-19 requiring enteral nutrition, which had an average BMI of 22.8 ± 1.9. Ultrasound images of 9 patients showed dynamic hyperechogenic area or 2 parallel hyperechogenic lines in the stomach. In one patient, we could find neither of these signs, and then the NGT was found coiled in the throat and mouth. After it was reset, ultrasound scanning confirmed the right position in stomach.

**Fig. 1:**
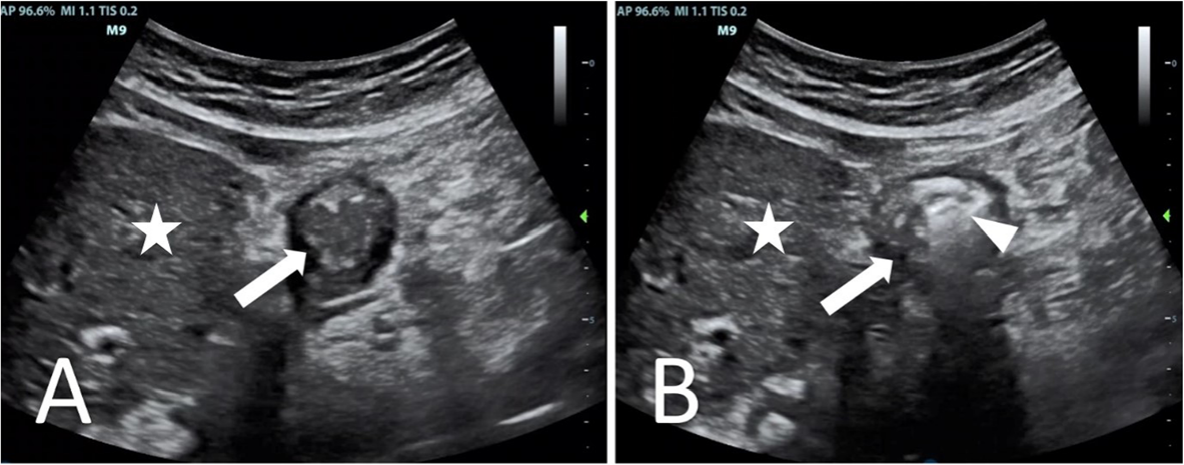
Male, 52 years old, with the chief complain “cough for 1 week and chest distress for 2 days.” A gastric tube was put. **a** Ultrasound showed the stomach (white arrow) and the liver (white star). **b** And then 20 ml of air was injected through the NGT; ultrasound showed dynamic hyperechogenic air shadow in the stomach (white triangle)

In our opinion, the use of ultrasound is essential in coupling with COVID-19 pandemic, especially lung ultrasound, which has been further confirmed [6]. We introduced another application of ultrasound during COVID-19 outbreaks, which might help to confirm the position of NGT in this special setting. There were some limitations in our report. The BMIs of the cases was not high, so it was easy for ultrasound observation. However, it might be difficult to find NGT by this way in obesity or flatulence patients, and the experience of operators could also influence the performance. And the sample of this report was small; a larger study is needed to verify the effectivity and safety of this method.

## Data Availability

The corresponding author had full access to the data and had final responsibility for the decision of submitting for publication.
